# Comparison of Submucosal With Intramuscular or Intravenous Administration of Dexamethasone for Third Molar Surgeries: A Systematic Review and Meta-Analysis

**DOI:** 10.3389/fsurg.2021.714950

**Published:** 2021-08-10

**Authors:** Chengyu Hou, Feng Liu, Chengbin Liu

**Affiliations:** Department of Oral and Maxillofacial Surgery, Zaozhuang Municipal Hospital, Zaozhuang, China

**Keywords:** third molar surgery, steroid, pain, inflammation, intravenous, intramuscular, submucosal

## Abstract

**Objective:** The study aimed to review evidence on the efficacy of submucosal (SM) administration vs. intravenous (IV) or intramuscular (IM) route of injections of dexamethasone for improving outcomes after mandibular third molar surgery.

**Methods:** PubMed, Embase, CENTRAL, and Google Scholar were searched for randomized controlled trials (RCTs) up to 20th May 2021. Early (2–3 days) and late (7 days) outcomes were compared between SM vs. IV or IM dexamethasone. Quality of evidence was assessed based on GRADE.

**Results:** Thirteen trials were included in the systematic review and 10 in the meta-analysis. Meta-analysis indicated a significant reduction in early pain with IV dexamethasone but no such difference for late pain compared to the SM group. There was no difference in early and late swelling scores between the SM and IV groups. Pooled analysis indicated no significant difference in early and late trismus between SM and IV groups. Comparing SM with IM dexamethasone, there was no significant difference in early and late pain scores. Swelling in the early and late postoperative periods was not significantly different between the two groups. There was no significant difference in early and late trismus between SM and IM groups. The quality of evidence was low for all outcomes.

**Conclusion:** Low-quality evidence suggests that SM infiltration of dexamethasone results in similar outcomes as compared to IV or IM administration of the drug after third molar surgeries. Further high-quality RCTs are needed to corroborate the current conclusions.

## Introduction

Mandibular third molar extraction is one of the most common surgical procedures in any oral surgical practice. A large number of mandibular third molars are impacted due to lack of adequate space for eruption and require surgical intervention for their removal (1). While many of the times, impacted mandibular third molars are asymptomatic, pathologies like recurrent pericoronitis, caries, and bone loss can necessitate early surgical extraction of these teeth (2). Research also indicates that asymptomatic third molars need to be extracted when the tooth is partially impacted in the horizontal or vertical position and with incomplete roots growing close to the mandibular canal (3).

Owing to the anatomical location of the impacted third molar, any surgical procedure leads to significant soft and hard tissue trauma (4). The surgical site around the third molar is surrounded by loose connective tissue of high vascularity and dense cortical bone. Adequate bone removal is required for safe delivery of the tooth and the degree of bone removal increases with the difficulty of the extraction (5). The tissue injury inflicted on account of the surgery results in an inflammatory response leading to significant pain, swelling, and edema in the postoperative period. The surgical procedure also affects the quality of life of the individual leading to impairment of routine activities (6).

Over the years, several interventions have been reported to reduce the postoperative sequelae of third molar surgery. Laser and piezoelectric instruments have been used as an alternative to rotary instruments for bone cutting (7). A variety of mouthwashes, antibiotics, analgesics, topical gels, cryotherapy, ozone therapy, and corticosteroids have also been used to reduce pain, swelling, and trismus after third molar surgeries (8). Of these various agents, the use of corticosteroids, especially dexamethasone is a popular intervention to improve outcomes of third molar surgery (9, 10).

In conventional surgical practice, dexamethasone is routinely administered via intravenous (IV) or intramuscular (IM) route. However, for a conscious patient undergoing mandibular third molar surgery, injection at a distal site can lead to added discomfort. Administration of the drug as a submucosal (SM) injection in the buccal vestibule at the surgical site has been suggested as an alternative (11). It is postulated that local infiltration of dexamethasone at the surgical site would have a more profound anti-inflammatory effect as compared to distal site parenteral administration (12). However, whether this theory holds in the case of mandibular third molar surgeries is still unclear. Over the last decade, many systematic reviews have provided evidence that administration of dexamethasone does lead to improved outcomes following third molar surgeries (13–16). However, to the best of our knowledge, no meta-analysis has evaluated the influence of the local vs. distal route of administration of dexamethasone on postoperative outcomes. In this context, The current study was designed to systematically review the evidence and conduct a meta-analysis to compare outcomes of surgical site infiltration (SM) vs. distal site parenteral administration (IV or IM) of dexamethasone to improve outcomes of patients undergoing mandibular third molar surgeries.

## Materials and Methods

This review was conducted as per the PRISMA statement (Preferred Reporting Items for Systematic Reviews and Meta-analyses) (17) and the Cochrane Handbook for Systematic Reviews of Intervention (18). The study was not registered on any online database. We aimed to answer the following research question: Is there a difference in postoperative outcomes of pain, swelling, and trismus with SM vs. IV or IM administration of dexamethasone in patients undergoing mandibular third molar surgeries?

### Literature Search

We conducted a systematic search of literature with the aid of a medical librarian. The databases of PubMed, Embase, and CENTRAL were searched electronically. Gray literature was searched using Google Scholar. We also search ClinicalTrials.gov for any ongoing trial. The search was conducted using a combination of the following keywords: “dexamethasone,” “steroid,” “corticosteroid,” “wisdom tooth,” “dental extraction,” “dental surgery,” “mandibular molar,” and “third molar” for all databases. Details are provided in Supplementary Table 1. Two reviewers (C.H. and F.L) carried out the electronic search independent of each other. We deduplicated the search results using Endnote (X9, Clarivate Analytics). The search results were assessed initially by their titles and abstracts to identify citations requiring full-text analysis. The full texts of the articles were reviewed by the two reviewers independently based on the inclusion and exclusion criteria. Any disagreements were resolved by discussion with the third reviewer (C.L.). For studies without abstract, we directly analyzed full-texts. Furthermore, we also hand-searched the bibliography of included studies for any missed references.

### Eligibility Criteria

We framed the inclusion criteria of this study based on the PICOS (Population, Intervention, Comparison, Outcome, and Study design) framework. Each domain was described as follows:

*Population*: Patients undergoing mandibular third molar surgeries
*Intervention*: SM dexamethasone
*Comparison*: IV or IM dexamethasone. IM injections were to be given in gluteal or deltoid regions.
*Outcomes*: Any one of the following: Pain, swelling, or trismus.
*Study design*: Randomized controlled trials (RCTs) only

Exclusion criteria were: (1) Studies using any other steroid drug like methylprednisolone (2) Studies on non-surgical mandibular third molar extractions or studies on maxillary third molar extractions (3) Studies on combined maxillary and mandibular third molar extractions (4) Studies conducted on patients taking dexamethasone preoperatively (5) Studies comparing SM with intramassetric injections only (6) Non-RCTs and uncontrolled studies. (7) Studies not reporting relevant outcomes (8) Editorials, review articles, and non-English language studies.

### Data Extraction and Risk of Bias Assessment

Two reviewers extracted data independently using a data extraction sheet. Data regarding the first author, publication year, study location, age and gender of the sample, sample size, type of impaction included, the dose of dexamethasone, post-operative mouthwash, antibiotics and analgesics, study outcomes, and follow-up were extracted. The aim was to compare pain, swelling, and trismus outcomes between SM vs. IV and SM vs. IM groups. Outcomes measured on postoperative day 2 or 3 were defined as early outcomes while those measured on day 7 were defined as late outcomes.

We used the recent Cochrane Collaboration's risk of bias assessment tool-2 to assess the quality of included RCTs (18). This was done by two reviewers independently. The following five domains were used for quality assessment: randomization process, deviation from intended intervention, missing outcome data, measurement of outcomes, and selection of reported result. Based on the risk of bias in individual domains, the overall bias was marked as “high risk,” “some concerns,” or “low risk.” Any disagreements related to data extraction or quality assessment were resolved by discussion. We also assessed the certainty of the evidence using the Grading of Recommendations Assessment, Development, and Evaluation (GRADE) tool using the GRADEpro GDT software [GRADEpro Guideline Development Tool. McMaster University, 2020 (developed by Evidence Prime, Inc.)].

### Statistical Analysis

“Review Manager” [RevMan, version 5.3; Nordic Cochrane Centre (Cochrane Collaboration), Copenhagen, Denmark; 2014] was used for the meta-analysis. We normalized the pain scores on a 10-point scale. Only final pain scores were pooled for the meta-analysis. Some studies reported a change in swelling and mouth opening scores while others reported only changed scores (difference between post-operative and pre-operative values). Since pre-operative mouth opening data was available from all included studies, we converted final mouth opening scores to change in mouth opening scores based on the Cochrane methodology (18) and pooled them for a meta-analysis. However, since preoperative facial measurements were not available from all studies, we pooled final scores and change scores separately for the outcome of swelling.

Data reported on the same scale (pain and trismus) was summarized using mean difference (MD) with 95% confidence intervals (CI) while swelling data was summarized using standardized mean difference (SMD) as there were variations in the methods used by the included studies. For studies reporting data only in graphical format, Engauge Digitizer Version 12.1 was used to extract data. Median, range, and interquartile range data was converted into mean and standard deviation (SD) when required using the method of Wan et al. (19). The random-effects model was used for all the meta-analyses. Heterogeneity was assessed using the *I*^2^ statistic. *I*^2^ values of 25–50% represented low, values of 50–75% medium, and more than 75% represented substantial heterogeneity. As the total number of studies included in each meta-analysis was <10, funnel plots were not used to assess publication bias.

## Results

**Figure 1:**
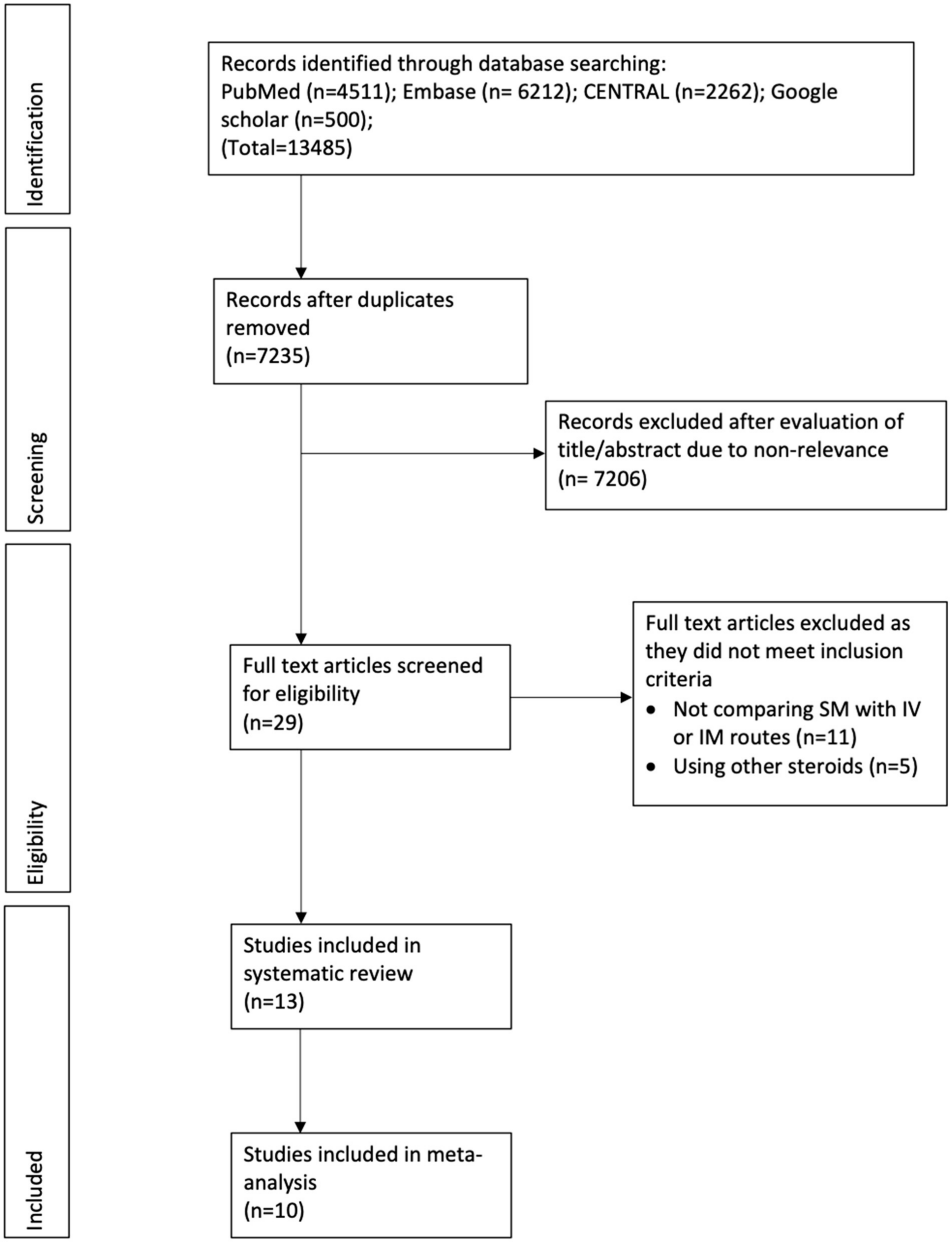
Study flow chart.

The search revealed a total of 7,235 unique records (Figure 1). A total of 29 articles were screened by their full texts of which 16 were excluded with reasons. Finally, 13 articles were included in the review (20–32).

Characteristics of included studies are presented in Table 1. The majority of studies were conducted in India followed by three trials in Iraq (22, 24, 32) and one each in Egypt (21) and New Zealand (28). The sample size of the included studies varied from 10 patients per arm to up to 65 patients per arm. Seven trials (22–24, 26, 30–32) used a dose of 4 mg of dexamethasone while others used 8 mg. However, the dose of dexamethasone was the same in the intervention and comparative arms of each trial. Three of the included trials were three-arm trials comparing the SM route with both IV and IM routes (23, 25, 32). Eight studies reported the use of chlorhexidine mouthwash postoperatively (22, 24, 27–32). The follow-up duration was 7 days in all trials. Outcome data were not reported as mean and SD by three studies and these were not included in the meta-analysis (20, 30, 31).

**Table 1:** Details of included studies.

**References**	**Study location**	**Age (years)**	**Gender**	Impaction type^*^	**Sample size**	**Dosage of dexamethasone**	**Site of IM injection**	**Mouth rinse used**	**Antibiotics used**	**Analgesic used**	**Follow-up**
Ramadan (21)	Egypt	18–40	19 M, 11 F	NR	SC: 15 IM: 15	8 mg 8 mg	NR	NR	Amoxicillin 500 mg TDS for 5 days	PCM 500 mg TDS for 3 days	7 days
Vivek et al. (25)	India	18−45	NR	Class II Type B	SC: 15 IV: 15	8 mg 8 mg	–	NR	Amoxicillin 500 mg TDS for 5 days	PCM 500 mg TDS for 3 days	7 days
Sreesha et al. (26)	India	18–45	NR	NR	SC: 32 IV: 32	4 mg 4 mg	–	NR	NR	NR	7 days
Hiriyanna and Degala (27)	India	18–40	20 M, 10 F	Class II/III Type A/B	SC: 16 IV: 17	8 mg 8 mg	–	0.012% Chlorhexidine	Amoxicillin 500 mg TDS for 5 days	Tramadol for 2 days thereafter PCM	7 days
Lau et al. (28)	New Zealand	16-40	57 M, 75 F	NR	SC: 65 IV: 65	8 mg 8 mg	–	0.2% Chlorhexidine	NR	PCM 1,000 mg QID, Ibuprofen 400 mg QID, codeine 30-60 mg QID	7 days
Agrawal et al. (20)	India	20-40	12 M, 18 F	NR	SC: 15 IV: 15	8 mg 8 mg	–	NR	Amoxicillin 500 mg TDS, Metronidazole 400 mg TDS for 5 days	PCM 325 mg and Ibuprofen 400 mg TDS for 3 days	7 days
Sahore and Parmar (29)	India	20-35	16 M, 4 F	Class II/III Type A/B	SC: 10 IM: 10	8 mg 8 mg	Deltoid	0.2% Chlorhexidine	Amoxicillin 500 mg plus clavulanic acid 125 mg TDS for 5 days	Diclofenac	7 days
Gopinath et al. (30)	India	>18	NR	NR	SC: 40 IV: 40	4 mg 4 mg	–	0.2% Chlorhexidine	Amoxicillin 2 g before surgery	NR	7 days
Gopalkrishnan et al. (31)	India	20–50	18 M, 42 F	NR	SC: 30 IM: 30	4 mg 4 mg	Deltoid	Chlorhexidine	Amoxicillin 500 mg plus clavulanic acid 125 mg TDS for 3 days	NR	7 days
Majid and Mahmood (32)	Iraq	18–48	16 M, 19 F	Class II/III Type A/B/C	SC: 11 IV: 12 IM: 12	4 mg 4 mg 4 mg	Deltoid	Chlorhexidine	Amoxicillin 500 mg TDS for 5 days	Tramadol 50 mg as rescue analgesic	7 days
Bhargava and Bureau (23)	India	19–30	NR	Class II Type B	SC: 10 IV: 10 IM: 10	4 mg 4 mg 4 mg	Deltoid	NR	Amoxicillin 500 mg TDS for 5 days	PCM 650 mg TDS for 5 days	7 days
Majid (22)	Iraq	19–48	12 M, 10 F	Class II/III Type B/C	SC: 11 IM: 11	4 mg 4 mg	NR	Chlorhexidine	Amoxicillin 500 mg TDS for 5 days	Tramadol 50 mg as rescue analgesic	7 days
Majid and Mahmood (24)	Iraq	20–48	12 M, 8 F	Class II/III Type A/B/C	SC: 10 IM: 10	4 mg 4 mg	NR	Chlorhexidine	Amoxicillin 500 mg TDS for 5 days	Tramadol 50 mg as rescue analgesic	7 days

* *Pell and Gregory classification*.

*SC, Submucosal; IV, intravenous; IM, intramuscular; M, male; F, female; PCM, paracetamol; NR, not reported; TDS, thrice daily; QID, four times daily*.

The risk of bias assessment of included studies is presented in Table 2. The risk of bias was the same for all outcomes and hence only one assessment is presented. Overall only three studies had a low risk of bias (24, 27, 28), one study (32) had some concerns while all the remaining studies had a high risk of bias.

**Table 2:** Risk of bias in included studies.

**Study**	**Randomization process**	**Deviation from intended intervention**	**Missing outcome data**	**Measurement of outcomes**	**Selection of reported result**	**Overall risk of bias**
Ramadan (21)	High risk	High risk	High risk	Some concerns	Low risk	High risk
Vivek et al. (25)	High risk	High risk	High risk	Some concerns	Low risk	High risk
Sreesha et al. (26)	High risk	High risk	High risk	Some concerns	Low risk	High risk
Hiriyanna and Degala (27)	Low risk	Low risk	Low risk	Low risk	Low risk	Low risk
Lau et al. (28)	Low risk	Low risk	Low risk	Low risk	Low risk	Low risk
Agrawal et al. (20)	High risk	High risk	High risk	Some concerns	High risk	High risk
Sahore and Parmar (29)	Low risk	High risk	High risk	Some concerns	Low risk	High risk
Gopinath et al. (30)	High risk	High risk	High risk	Some concerns	Low risk	High risk
Gopalkrishnan et al. (31)	High risk	High risk	High risk	Some concerns	Low risk	High risk
Majid and Mahmood (32)	Low risk	Low risk	Low risk	Some concerns	Low risk	Some concerns
Bhargava and Bureau (23)	High risk	Low risk	High risk	Low risk	Low risk	High risk
Majid (22)	Low risk	Low risk	Low risk	Low risk	Low risk	Low risk
Majid and Mahmood (24)	Low risk	Low risk	High risk	Low risk	Low risk	High risk

### SM vs. IV Dexamethasone

**Figure 2:**
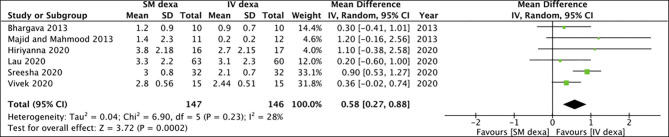
Meta-analysis of early pain between SM and IV dexamethasone (dexa) for third molar surgeries.

**Figure 3:**
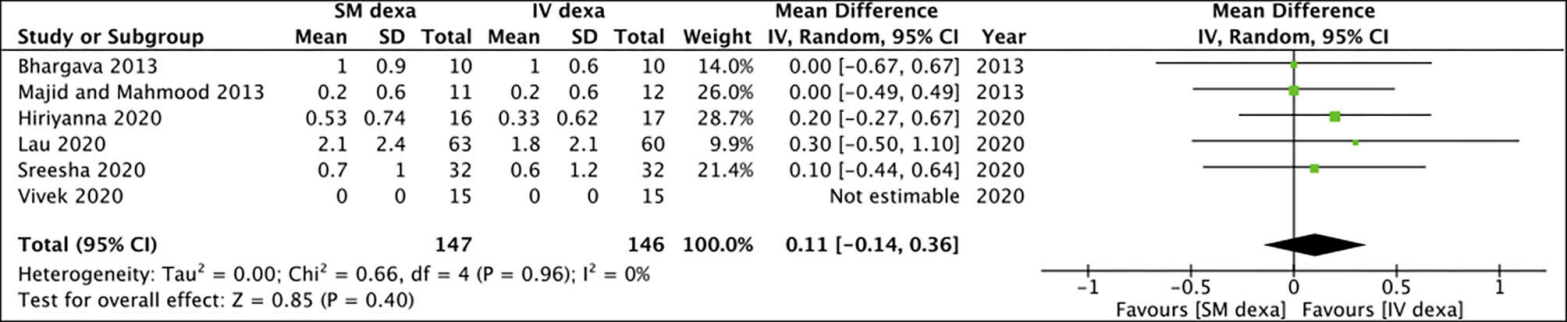
Meta-analysis of late pain between SM and IV dexamethasone (dexa) for third molar surgeries.

Six studies compared SM with IV dexamethasone. Meta-analysis indicated a statistically significant reduction in early pain scores in patients receiving IV dexamethasone compared to those receiving SM dexamethasone (MD: 0.58 95% CI: 0.27, 0.88 *I*^2^ = 28% *p* = 0.0002) (Figure 2). However, no such difference was noted between the two groups for late pain scores (MD: 0.11 95% CI: −0.14 to 0.36 *I*^2^ = 0% *p* = 0.40) (Figure 3).

**Figure 4:**
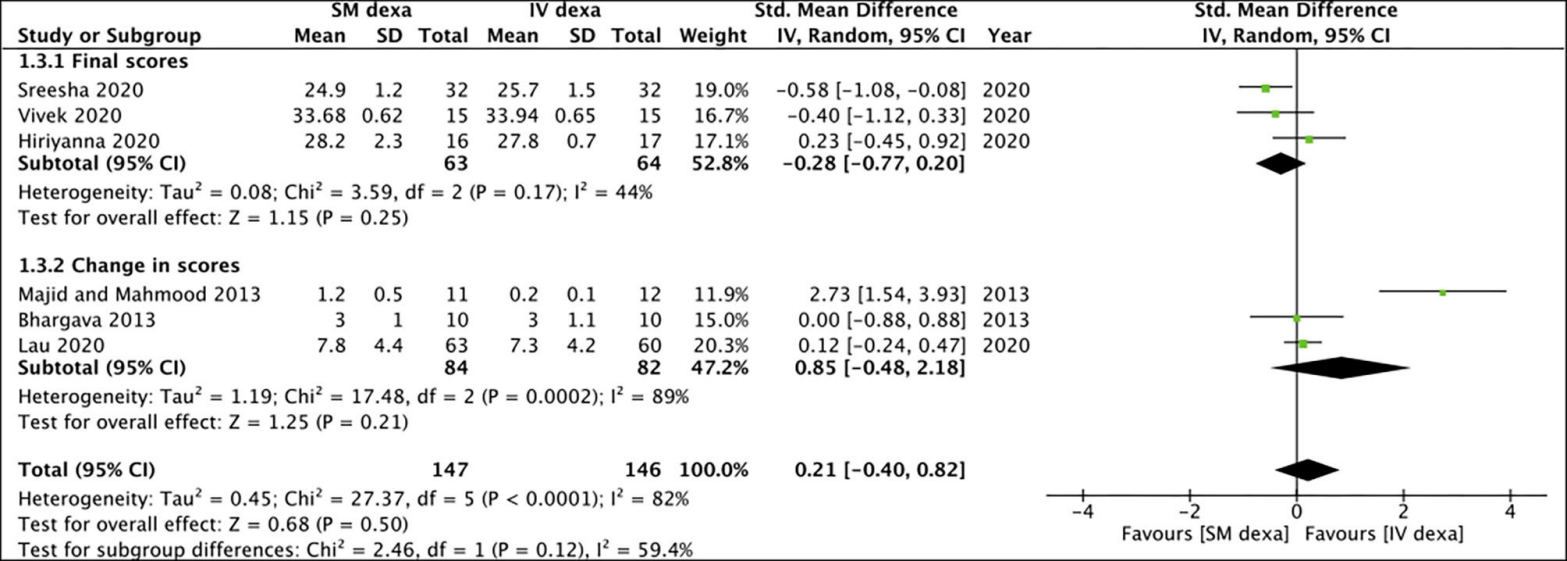
Meta-analysis of early swelling between SM and IV dexamethasone (dexa) for third molar surgeries. Sub-group analysis performed based on final scores or change scores.

**Figure 5:**
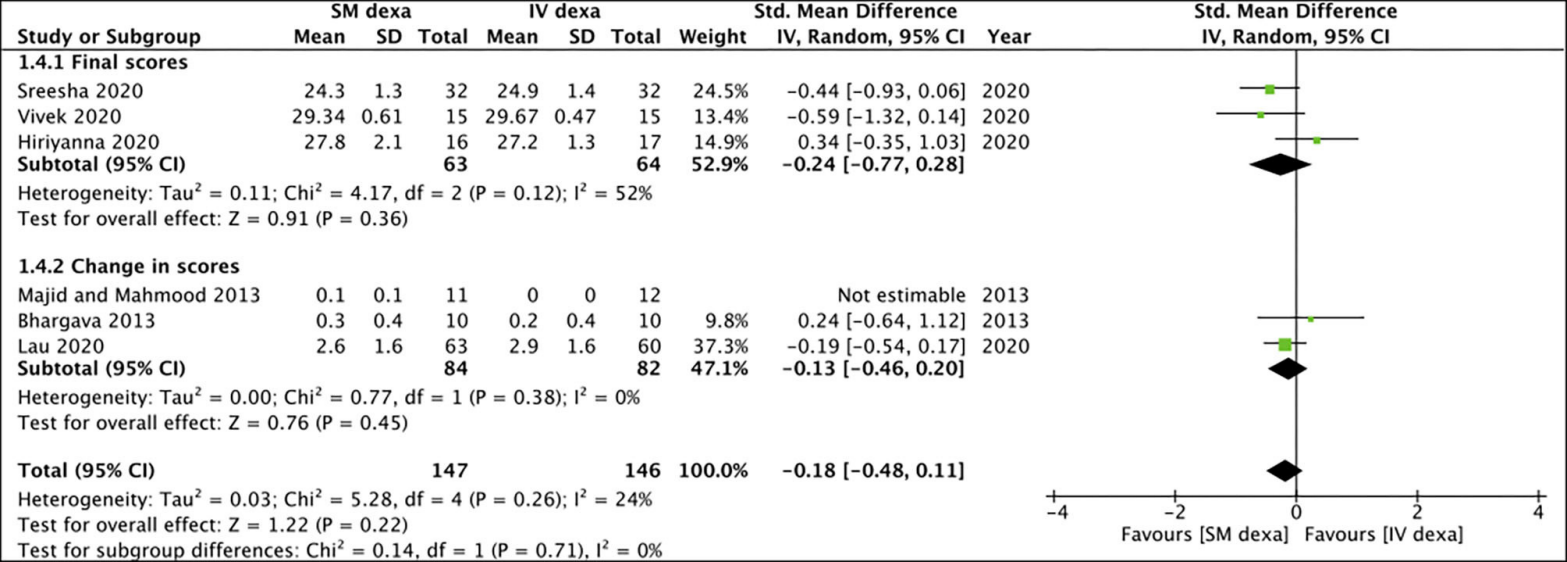
Meta-analysis of late swelling between SM and IV dexamethasone (dexa) for third molar surgeries. Sub-group analysis performed based on final scores or change scores.

Of the six studies, three trials reported change scores for the outcome of swelling while the remaining reported final scores. Meta-analysis demonstrated no statistically significant difference in final swelling scores (SMD: −0.28 95% CI: −0.77 to 0.20 *I*^2^ = 44% *p* = 0.25) as well as change in swelling scores (SMD: 0.85 95% CI: −0.48 to 2.18 *I*^2^ = 89% *p* = 0.21) between SM and IV groups in the early postoperative period (Figure 4). Similar results were noted for late swelling outcomes for both final (SMD:−0.24 95% CI: −0.77 to 0.28 *I*^2^ = 52% *p* = 0.36) and change scores (SMD: −0.13 95% CI: −0.46 to 0.20 *I*^2^ = 0% *p* = 0.45) (Figure 5).

**Figure 6:**
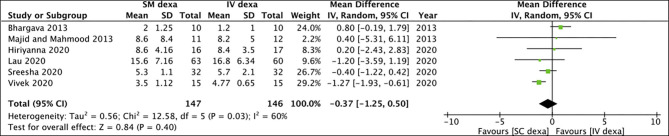
Meta-analysis of early trismus between SM and IV dexamethasone (dexa) for third molar surgeries.

**Figure 7:**
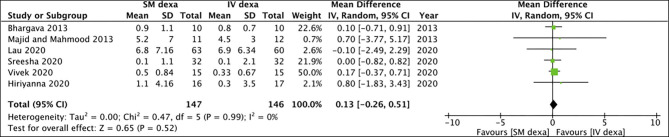
Meta-analysis of late trismus between SM and IM dexamethasone (dexa) for third molar surgeries.

Pooled analysis of all six studies indicated no statistically significant difference in early trismus between SM and IV dexamethasone groups (MD: −0.37 95% CI: −1.25 to 0.50 *I*^2^ = 60% *p* = 0.40) (Figure 6). Similar non-significant results were noted for late trismus outcomes as well (MD: 0.13 95% CI: −0.26 to 0.51 *I*^2^ = 0% *p* = 0.52) (Figure 7). GRADE assessment of evidence for SM vs. IV dexamethasone is presented in Supplementary Table 2. Owing to the high risk of bias in majority studies, the quality of evidence was deemed to be low for all outcomes.

### SM vs. IM Dexamethasone

**Figure 8:**
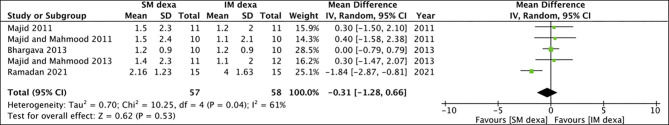
Meta-analysis of early pain between SM and IM dexamethasone (dexa) for third molar surgeries.

**Figure 9:**
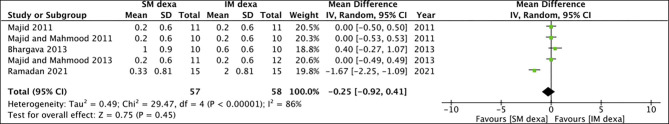
Meta-analysis of late pain between SM and IM dexamethasone (dexa) for third molar surgeries.

Of the seven studies included in the meta-analysis of SM vs. IM dexamethasone, five reported data on pain scores. Pooled analysis demonstrated no statistically significant difference in early (MD: −0.31 95% CI: −1.28 to 0.66 *I*^2^ = 61% *p* = 0.53) (Figure 8) as well as late pain scores (MD: −0.25 95% CI: −0.92 to 0.41 *I*^2^ = 86% *p* = 0.45) (Figure 9) between SM and IM groups.

**Figure 10:**
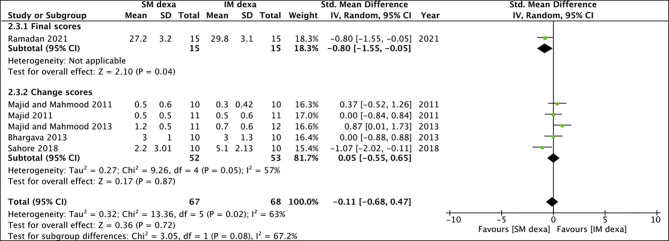
Meta-analysis of early swelling between SM and IM dexamethasone (dexa) for third molar surgeries. Sub-group analysis performed based on final scores or change scores.

**Figure 11:**
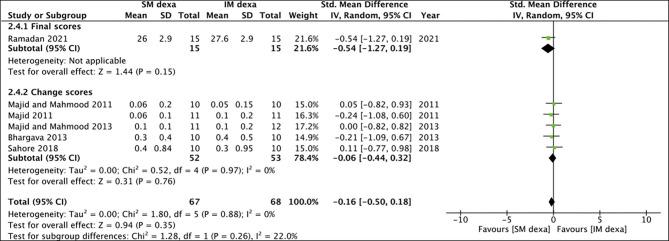
Meta-analysis of late swelling between SM and IM dexamethasone (dexa) for third molar surgeries. Sub-group analysis performed based on final scores or change scores.

Six studies reported data on swelling of which one reported final scores while the remaining reported change scores. On meta-analysis of final swelling scores, we noted a small significant difference in early swelling between SM and IM dexamethasone groups (SMD: −0.80 95% CI: −1.55 to −0.05 *p* = 0.04) (Figure 10). However, non-significant results were obtained on analysis of change scores for early swelling between the two groups (SMD: 0.05 95% CI: −0.55, 0.65 *I*^2^ = 57% *p* = 0.87). Pooled analysis also failed to demonstrate any statistically significant difference in late swelling for studies reporting final scores (SMD: −0.54 95% CI: −1.27 to 0.19 *p* = 0.15) as well as for those reporting change scores (SMD: −0.06 95% CI: −0.44 to 0.32 *I*^2^ = 0% *p* = 0.76) (Figure 11).

**Figure 12:**
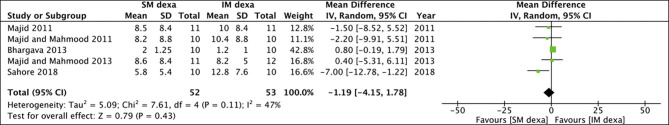
Meta-analysis of early trismus between SM and IM dexamethasone (dexa) for third molar surgeries.

**Figure 13:**
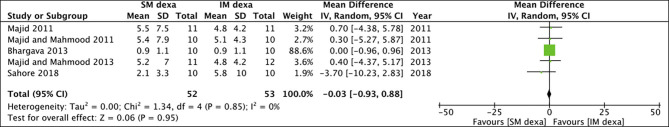
Meta-analysis of late trismus between SM and IM dexamethasone (dexa) for third molar surgeries.

Six studies reported data on trismus. Meta-analysis indicated no statistically significant difference in early (MD: −1.19 95% CI: −4.15 to 1.78 *I*^2^ = 47% *p* = 0.43) (Figure 12) and late trismus (MD: −0.03 95% CI: −0.93 to 0.88 *I*^2^ = 0% *p* = 0.95) (Figure 13) outcomes between SM and IM dexamethasone. GRADE assessment of evidence for SM vs. IM dexamethasone is presented in Supplementary Table 3. Owing to the high risk of bias in majority studies, the quality of evidence was deemed to be low for all outcomes.

## Discussion

Our study which is the first systematic review and meta-analysis comparing outcomes of SM vs. IV or IM dexamethasone indicates that the route of administration has little impact on the outcomes of mandibular third molar surgery. We noted only a mild beneficial effect of IV dexamethasone as compared to SM dexamethasone for early pain. There were no statistically significant differences between late pain, swelling, and trismus outcomes between SM and IV or IM administration of dexamethasone.

Mandibular third molar surgeries can lead to significant postoperative morbidity due to pain, extraoral edema, and reduced mouth opening which can greatly hamper the quality of life of the patient. Consequently, administration of corticosteroids has become standard practice with third molar surgeries (9, 10). Corticosteroids act by inhibiting the enzyme phospholipase A2 which reduces the production of arachidonic acid at the inflammation site. Since arachidonic acid is an important precursor for several inflammatory mediators, inhibition of its production results in an attenuated inflammatory response with a resultant effect on adverse postoperative signs and symptoms (33). As the action of corticosteroids is primarily related to the presence of tissue injury or inflammation, infiltration of the drug directly at the surgical site should have a repository effect with direct and prolonged action on the tissues. Local infiltration should result in slow absorption of the drug with a longer duration of action as compared to distal parenteral administration of the drug with IV or IM routes (12). Indeed, Gao et al. (12) in an RCT on patients undergoing tonsillectomy have shown that tissue site infiltration of dexamethasone results in reduced pain and time to food intake as compared to patients receiving IV dexamethasone. Li et al. (34) in a recent study have demonstrated that intra-articular dexamethasone results in improved pain and swelling outcomes as compared to IV administration in patients undergoing total knee arthroplasty. A trial on patients undergoing cesarean section has also noted reduced pain scores with local infiltration of dexamethasone as compared to IV administration (35).

The results of our systematic review and meta-analysis, however, differ from the outcomes seen in other surgical specialties. On pooled analysis of SM vs. IV route, we noted a significant reduction of early pain scores with IV dexamethasone as compared to SM injections, but no difference in late pain, early and late swelling as well as early and late trismus scores between the two groups. Comparing SM vs. IM route, there was no difference in any of the outcomes of interest. Important to note is that the effect size of pain scores with IV dexamethasone in the early postoperative period was very small. Significant reduction of pain score with IV dexamethasone by 0.58 points on a 10-point pain scale may not be relevant in clinical practice. Martin et al. (36) have demonstrated that a 2.5 point reduction of pain score on the Visual Analog Scale is considered to be clinically significant for third molar surgeries.

Considering the better outcomes reported with local infiltration of dexamethasone in other surgical fields, the lack of difference in outcomes between SM and distal routes of administration is difficult to explain in the context of third molar surgeries. One possible reason could be that the high vascularity of the third molar region which receives rich blood supply from the inferior alveolar, facial and lingual arteries leads to the rapid absorption of dexamethasone after SM injections and subsequent attenuation of the local effect of the drug (37). However, to substantiate this theory there is a need for studies comparing plasma concentrations of the drug following SM and IV or IM administration. Secondly, a major component of the surgical trauma during third molar surgery results from bone removal. Research suggests that minimizing heat generation during bone cutting using piezosurgery reduces osteonecrosis and maintains viable osteocytes. The reduced trauma during bone cutting results in substantial improvements in pain, swelling, and trismus outcomes after third molar surgery (7). Therefore, theoretically, SM administration of dexamethasone may not lead to a direct action on the bone which is a major source of inflammatory mediators after surgery. Indeed, a recent study has compared outcomes of SM vs. direct intra-osseous administration of dexamethasone for third molar surgeries but found no difference in pain and swelling between the two routes (11). With just a single trial to date, there is a need for further studies comparing SM and intra-osseous administration of dexamethasone to delineate evidence on the difference between the two routes.

Other than dexamethasone, corticosteroids like methylprednisolone (MP) and triamcinolone have also been used after third molar surgeries (10). However, there is a paucity of evidence on the influence of different routes of administration of other corticosteroid drugs on mandibular third molar surgeries. In a comprehensive review, Nagori et al. (38) have demonstrated the MP administered via any route results in a reduction of early postoperative edema. However, no comparative analysis between SM and IV or IM routes could be carried out due to a lack of studies. Mukund et al. (39) in a trial comparing oral, SM, IV, and intramasseteric administration of MP reported better outcomes with the intramasseteric route after third molar surgeries. On the other hand, Selvaraj et al. (40) did not find any difference in outcomes between intramasseteric and IM injections of MP at the gluteal region.

It can be noted that two different doses of dexamethasone (4 mg or 8 mg) were used in the included studies. However, since the dosage was the same for both the intervention and comparative arms, there would have been a minimal impact of different doses on postoperative outcomes. Furthermore, studies have demonstrated that the different doses of dexamethasone (4 mg, 8 mg, or 12 mg) do not lead to statistically significant differences in outcomes after third molar surgery (41, 42). We also noted differences in the type of impactions and type of analgesics amongst the included studies. However, with every included study being an RCT, we believe the type of impaction would not have significantly altered the results. However, a split-mouth study with similar type of impactions would have indeed provided better evidence. The type of analgesic used in the included studies was the same for both intervention and control groups and therefore the influence of this confounding factor would have been minimal.

Our study has some limitations. Firstly, despite a thorough literature search, only a limited number of RCTs were available for inclusion in the review. Due to differences in comparative groups, only a small number of studies could be included in each meta-analysis. Moreover, the sample size of many studies was small and the total number of patients analyzed despite a pooled analysis was not very high. Secondly, there were only three studies with a low risk of bias (24, 27, 28). There were concerns regarding randomization, allocation concealment, and blinding of outcome measures in many studies. This may have introduced significant bias in the outcomes of the included trials. Consequently, the overall quality of evidence was deemed to be low for all our comparisons. Thirdly, the majority of the trials were from just two countries. This limits the generalizability of evidence from this meta-analysis.

## Conclusion

Low-quality evidence suggests that SM infiltration of dexamethasone results in similar outcomes as compared to IV or IM administration of the drug after third molar surgeries. In clinical practice, surgeons may be more comfortable with the SM route as infiltration is simple and the drug is injected at the anesthetized surgical site. However, further high-quality RCTs are needed to corroborate the current conclusions. Future trials should be multi-centric and of large sample size to strengthen generalizability of results. The trials should also ensure adequate randomization, allocation concealment, and blinding of outcome measures to generate high-quality evidence.

## Data Availability Statement

The raw data supporting the conclusions of this article will be made available by the authors, without undue reservation.

## Author Contributions

CH, FL, and CL conceived and designed the study. CH and FL were involved in literature search, data collection, analyzed the data, reviewed, and edited the manuscript. FL and CL wrote the paper. All authors contributed to the article and approved the submitted version.

## Conflict of Interest

The authors declare that the research was conducted in the absence of any commercial or financial relationships that could be construed as a potential conflict of interest.

## Publisher's Note

All claims expressed in this article are solely those of the authors and do not necessarily represent those of their affiliated organizations, or those of the publisher, the editors and the reviewers. Any product that may be evaluated in this article, or claim that may be made by its manufacturer, is not guaranteed or endorsed by the publisher.
