# Supplementary material for: Comparison of Submucosal With Intramuscular or Intravenous Administration of Dexamethasone for Third Molar Surgeries: A Systematic Review and Meta-Analysis
Source: Front Surg. 2021 Aug 10;8:714950. doi: 10.3389/fsurg.2021.714950 (PMC8382880; doi:10.3389/fsurg.2021.714950)
Supplement: Supplementary file 1 [file Table_1.DOCX]

Supplementary Table 1:Search strategy

| **Query** | **Search Details** |
| --- | --- |
| (((dexamethasone) OR (steroid)) OR (corticosteroid)) AND (mandibular molar) | ("dexamethason"[All Fields] OR "dexamethasone"[MeSH Terms] OR "dexamethasone"[All Fields] OR "dexamethasone s"[All Fields] OR "dexamethasones"[All Fields] OR ("steroidal"[All Fields] OR "steroidals"[All Fields] OR "steroidic"[All Fields] OR "steroids"[MeSH Terms] OR "steroids"[All Fields] OR "steroid"[All Fields]) OR ("adrenal cortex hormones"[MeSH Terms] OR ("adrenal"[All Fields] AND "cortex"[All Fields] AND "hormones"[All Fields]) OR "adrenal cortex hormones"[All Fields] OR "corticosteroid"[All Fields] OR "corticosteroids"[All Fields] OR "corticosteroidal"[All Fields] OR "corticosteroide"[All Fields] OR "corticosteroides"[All Fields])) AND (("mandible"[MeSH Terms] OR "mandible"[All Fields] OR "mandibular"[All Fields] OR "mandibulars"[All Fields]) AND ("molar"[MeSH Terms] OR "molar"[All Fields] OR "molars"[All Fields] OR "molar s"[All Fields])) |
| (((dexamethasone) OR (steroid)) OR (corticosteroid)) AND (third molar) | ("dexamethason"[All Fields] OR "dexamethasone"[MeSH Terms] OR "dexamethasone"[All Fields] OR "dexamethasone s"[All Fields] OR "dexamethasones"[All Fields] OR ("steroidal"[All Fields] OR "steroidals"[All Fields] OR "steroidic"[All Fields] OR "steroids"[MeSH Terms] OR "steroids"[All Fields] OR "steroid"[All Fields]) OR ("adrenal cortex hormones"[MeSH Terms] OR ("adrenal"[All Fields] AND "cortex"[All Fields] AND "hormones"[All Fields]) OR "adrenal cortex hormones"[All Fields] OR "corticosteroid"[All Fields] OR "corticosteroids"[All Fields] OR "corticosteroidal"[All Fields] OR "corticosteroide"[All Fields] OR "corticosteroides"[All Fields])) AND ("molar, third"[MeSH Terms] OR ("molar"[All Fields] AND "third"[All Fields]) OR "third molar"[All Fields] OR ("third"[All Fields] AND "molar"[All Fields])) |
| (((dexamethasone) OR (steroid)) OR (corticosteroid)) AND (dental surgery) | ("dexamethason"[All Fields] OR "dexamethasone"[MeSH Terms] OR "dexamethasone"[All Fields] OR "dexamethasone s"[All Fields] OR "dexamethasones"[All Fields] OR ("steroidal"[All Fields] OR "steroidals"[All Fields] OR "steroidic"[All Fields] OR "steroids"[MeSH Terms] OR "steroids"[All Fields] OR "steroid"[All Fields]) OR ("adrenal cortex hormones"[MeSH Terms] OR ("adrenal"[All Fields] AND "cortex"[All Fields] AND "hormones"[All Fields]) OR "adrenal cortex hormones"[All Fields] OR "corticosteroid"[All Fields] OR "corticosteroids"[All Fields] OR "corticosteroidal"[All Fields] OR "corticosteroide"[All Fields] OR "corticosteroides"[All Fields])) AND (("dental health services"[MeSH Terms] OR ("dental"[All Fields] AND "health"[All Fields] AND "services"[All Fields]) OR "dental health services"[All Fields] OR "dental"[All Fields] OR "dentally"[All Fields] OR "dentals"[All Fields]) AND ("surgery"[MeSH Subheading] OR "surgery"[All Fields] OR "surgical procedures, operative"[MeSH Terms] OR ("surgical"[All Fields] AND "procedures"[All Fields] AND "operative"[All Fields]) OR "operative surgical procedures"[All Fields] OR "general surgery"[MeSH Terms] OR ("general"[All Fields] AND "surgery"[All Fields]) OR "general surgery"[All Fields] OR "surgery s"[All Fields] OR "surgerys"[All Fields] OR "surgeries"[All Fields])) |
| (((dexamethasone) OR (steroid)) OR (corticosteroid)) AND (dental extraction) | ("dexamethason"[All Fields] OR "dexamethasone"[MeSH Terms] OR "dexamethasone"[All Fields] OR "dexamethasone s"[All Fields] OR "dexamethasones"[All Fields] OR ("steroidal"[All Fields] OR "steroidals"[All Fields] OR "steroidic"[All Fields] OR "steroids"[MeSH Terms] OR "steroids"[All Fields] OR "steroid"[All Fields]) OR ("adrenal cortex hormones"[MeSH Terms] OR ("adrenal"[All Fields] AND "cortex"[All Fields] AND "hormones"[All Fields]) OR "adrenal cortex hormones"[All Fields] OR "corticosteroid"[All Fields] OR "corticosteroids"[All Fields] OR "corticosteroidal"[All Fields] OR "corticosteroide"[All Fields] OR "corticosteroides"[All Fields])) AND ("tooth extraction"[MeSH Terms] OR ("tooth"[All Fields] AND "extraction"[All Fields]) OR "tooth extraction"[All Fields] OR ("dental"[All Fields] AND "extraction"[All Fields]) OR "dental extraction"[All Fields]) |
| (((dexamethasone) OR (steroid)) OR (corticosteroid)) AND (wisdom tooth) | ("dexamethason"[All Fields] OR "dexamethasone"[MeSH Terms] OR "dexamethasone"[All Fields] OR "dexamethasone s"[All Fields] OR "dexamethasones"[All Fields] OR ("steroidal"[All Fields] OR "steroidals"[All Fields] OR "steroidic"[All Fields] OR "steroids"[MeSH Terms] OR "steroids"[All Fields] OR "steroid"[All Fields]) OR ("adrenal cortex hormones"[MeSH Terms] OR ("adrenal"[All Fields] AND "cortex"[All Fields] AND "hormones"[All Fields]) OR "adrenal cortex hormones"[All Fields] OR "corticosteroid"[All Fields] OR "corticosteroids"[All Fields] OR "corticosteroidal"[All Fields] OR "corticosteroide"[All Fields] OR "corticosteroides"[All Fields])) AND ("molar, third"[MeSH Terms] OR ("molar"[All Fields] AND "third"[All Fields]) OR "third molar"[All Fields] OR ("wisdom"[All Fields] AND "tooth"[All Fields]) OR "wisdom tooth"[All Fields]) |
