# Supplementary material for: Comparison of Submucosal With Intramuscular or Intravenous Administration of Dexamethasone for Third Molar Surgeries: A Systematic Review and Meta-Analysis
Source: Front Surg. 2021 Aug 10;8:714950. doi: 10.3389/fsurg.2021.714950 (PMC8382880; doi:10.3389/fsurg.2021.714950)
Supplement: Supplementary file 2 [file Table_2.DOCX]

| **Supplementary Table 2: GRADE assessment of outcomes of submucosal vs intravenous dexamethasone for third molar surgery** | | | | | | | | | | | |
| --- | --- | --- | --- | --- | --- | --- | --- | --- | --- | --- | --- |
| **Certainty assessment** | | | | | | | **Summary of findings** | | | | |
| **Participants  (studies) Follow up** | **Risk of bias** | **Inconsistency** | **Indirectness** | **Imprecision** | **Publication bias** | **Overall certainty of evidence** | **Study event rates (%)** | | **Relative effect (95% CI)** | **Anticipated absolute effects** | |
|  |  |  |  |  |  |  | **With IV dexa** | **With SM dexa** |  | **Risk with IV dexa** | **Risk difference with SM dexa** |
| **Early Pain** | | | | | | | | | | | |
| 293 (6 RCTs) | very serious ^a^ | not serious | not serious | not serious | none | ⨁⨁◯◯ LOW | 146 | 147 | - | The mean pain SM vs IV early was **0** | MD **0.58 higher** (0.27 higher to 0.88 higher) |
| **Late** **Pain** | | | | | | | | | | | |
| 293 (6 RCTs) | very serious ^a^ | not serious | not serious | not serious | none | ⨁⨁◯◯ LOW | 146 | 147 | - | The mean pain SM vs IV late was **0** | MD **0.11 higher** (0.14 lower to 0.36 higher) |
| **Early Swelling** | | | | | | | | | | | |
| 293 (6 RCTs) | very serious ^a^ | not serious | not serious | not serious | none | ⨁⨁◯◯ LOW | 146 | 147 | - | - | SMD **0.21 higher** (0.4 lower to 0.82 higher) |
| **Late Swelling** | | | | | | | | | | | |
| 293 (6 RCTs) | very serious ^a^ | not serious | not serious | not serious | none | ⨁⨁◯◯ LOW | 146 | 147 | - | - | SMD **0.18 lower** (0.48 lower to 0.11 higher) |
| **Early Trismus** | | | | | | | | | | | |
| 293 (6 RCTs) | very serious ^a^ | not serious | not serious | not serious | none | ⨁⨁◯◯ LOW | 146 | 147 | - | The mean early trismus SM vs IV was **0** | MD **0.37 lower** (1.25 lower to 0.5 higher) |
| **Late Trismus** | | | | | | | | | | | |
| 293 (6 RCTs) | very serious ^a^ | not serious | not serious | not serious | none | ⨁⨁◯◯ LOW | 146 | 147 | - | The mean late trismus SM vs IV was **0** | MD **0.13 higher** (0.26 lower to 0.51 higher) |

**CI:** Confidence interval; **MD:** Mean difference; **SMD:** Standardised mean difference; **IV**, intravenous; **SM**: Submucosal; **Dexa**: Dexamethasone

#### Explanations

a. High overall risk of bias in the studies of Vivek 2020, Sreesha 2020, Bhargava 2013
