# Supplementary material for: Comparison of Submucosal With Intramuscular or Intravenous Administration of Dexamethasone for Third Molar Surgeries: A Systematic Review and Meta-Analysis
Source: Front Surg. 2021 Aug 10;8:714950. doi: 10.3389/fsurg.2021.714950 (PMC8382880; doi:10.3389/fsurg.2021.714950)
Supplement: Supplementary file 3 [file Table_3.DOCX]

| **Supplementary Table 3: GRADE assessment of outcomes with submucosal vs intramuscular dexamethasone for third molar surgery** | | | | | | | | | | | |
| --- | --- | --- | --- | --- | --- | --- | --- | --- | --- | --- | --- |
| **Certainty assessment** | | | | | | | **Summary of findings** | | | | |
| **Participants  (studies) Follow up** | **Risk of bias** | **Inconsistency** | **Indirectness** | **Imprecision** | **Publication bias** | **Overall certainty of evidence** | **Study event rates (%)** | | **Relative effect (95% CI)** | **Anticipated absolute effects** | |
|  |  |  |  |  |  |  | **With IM dexa** | **With SM dexa** |  | **Risk with IM dexa** | **Risk difference with SM dexa** |
| **Early** **Pain** | | | | | | | | | | | |
| 115 (5 RCTs) | very serious ^a^ | not serious | not serious | not serious | none | ⨁⨁◯◯ LOW | 58 | 57 | - | The mean pain early was **0** | MD **0.31 lower** (1.28 lower to 0.66 higher) |
| **Late Pain** | | | | | | | | | | | |
| 115 (5 RCTs) | very serious ^a^ | not serious | not serious | not serious | none | ⨁⨁◯◯ LOW | 58 | 57 | - | The mean pain late was **0** | MD **0.25 lower** (0.92 lower to 0.41 higher) |
| **Early Swelling** | | | | | | | | | | | |
| 135 (6 RCTs) | very serious ^b^ | not serious | not serious | not serious | none | ⨁⨁◯◯ LOW | 68 | 67 | - | - | SMD **0.11 lower** (0.68 lower to 0.47 higher) |
| **Late Swelling** | | | | | | | | | | | |
| 135 (6 RCTs) | very serious ^b^ | not serious | not serious | not serious | none | ⨁⨁◯◯ LOW | 68 | 67 | - | - | SMD **0.16 lower** (0.50 lower to 0.18 higher) |
| **Early Trismus** | | | | | | | | | | | |
| 105 (5 RCTs) | very serious ^b^ | not serious | not serious | not serious | none | ⨁⨁◯◯ LOW | 53 | 52 | - | The mean trismus early was **0** | MD 1**.19 lower** (4.15 lower to 1.78 higher) |
| **Late Trismus** | | | | | | | | | | | |
| 105 (5 RCTs) | very serious ^b^ | not serious | not serious | not serious | none | ⨁⨁◯◯ LOW | 53 | 52 | - | The mean trismus late was **0** | MD **0.03 lower** (0.93 lower to 0.88 higher) |

**CI:** Confidence interval; **MD:** Mean difference; **SMD:** Standardised mean difference; **IM**, intramuscular; **SM**: Submucosal; **Dexa**: Dexamethasone

#### Explanations

a. High risk of overall bias in the studies of Ramadan 2021, Majid & Mahmood 2011.

b. High risk of overall bias in the studies of Ramadan 2021, Sahore 2018, Majid & Mahmood 2011.
